# Development of UV-Chemometric techniques for resolving the overlapped spectra of aspirin, caffeine and orphenadrine citrate in their combined pharmaceutical dosage form

**DOI:** 10.1186/s13065-025-01429-x

**Published:** 2025-03-20

**Authors:** Sobhy M. El-Adl, Amr A. Mattar, Omar M. El-Abassy, Mahmoud M. Sebaiy

**Affiliations:** 1 Medicinal Chemistry Department, Faculty of Pharmacy, Zagazig University, Zagazig, 44519 Egypt; 2 Pharmaceutical Chemistry Department, Faculty of Pharmacy, Egyptian Russian University, Badr City, Cairo 11829 Egypt

**Keywords:** Aspirin, Caffeine, Orphenadrine citrate, AGREE, BAGI, GAPI, PLS

## Abstract

A UV-chemometric approach has been developed to analyze a ternary combination of aspirin, caffeine, and orphenadrine citrate without the need for previous separation. The method is easy, specific, accurate, and highly precise. The three medications were evaluated simultaneously utilizing CLS, PLS, and PCR, which were generated based on separate data sets that yielded superior findings. Regrettably, their accurate estimation could only be achieved using the PLS approach. In order to determine the prediction power of each chemometric approach, its validity has been tested using 8 synthetic mixes. The latent variable number varies across various models as the dataset changes. The comparison of various methodologies and the assessment of the predictive capacity of each set of data were done using the predicted residual error sum of squares (PRESS) and the root mean square error of prediction (RMSEP). The created approach was also used to statistically compare the performance of PLS in a dataset with zero absorption, as well as to compare the performance of the offered chemometric methods in various datasets. The environmental impact of the created approach was assessed to determine the overall ecological sustainability of the designed methodology. According to the new Blue Applicability Grade Index (BAGI) evaluation methodology, the suggested technique was also found to be practicable.

**Supplementary Information:**

The online version contains supplementary material available at 10.1186/s13065-025-01429-x.

## Introduction

**Fig. 1:**
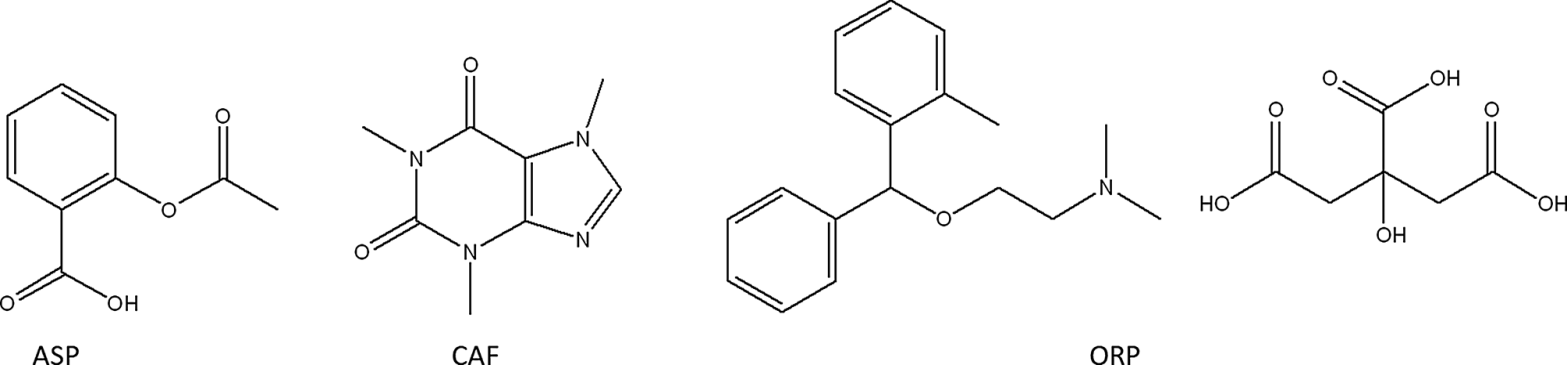
Chemical structures of aspirin (ASP), caffeine (CAF) and orphenadrine citrate (ORP)

Nonmalignant chronic pain (NMCP) is regarded as a matter of public health significance [1]. Every year in the United States, almost 100 million Americans suffer from chronic pain. The United States incurs an estimated added cost of $261-$300 billion in health care expenditure associated with chronic pain [2, 3]. The American Pain Society has advocated the use of polytherapy to achieve efficient pain management while minimizing the risk and occurrence of side effects [4]. The combination of aspirin (ASP), caffeine (CAF), and orphenadrine citrate (ORPH) is used to alleviate the discomfort associated with musculoskeletal pain. Acetylsalicylic acid, commonly known as aspirin (ASP), is a salicylate drug that rapidly proceeds hydrolysis in the human body to produce its therapeutic effects. It serves as a pain reliever, fever reducer, and anti-inflammatory medication (Fig. 1). ASP may also be utilized to decrease death from cardiovascular disease in high-risk individuals who have had a heart attack or stroke [5, 6]. The psychostimulant purine alkaloid caffeine (CAF), scientifically known as 1,3,7-trimethylxanthine, may heighten one’s state of alertness. (Fig. 1). It is common practice to mix CAF with analgesic and antipyretic medications because of its ability to enhance their effects [7]. Orphenadrine citrate (ORP) is a chemical compound with the chemical formula (±)-N, N-Dimethyl-2-[(o-methyl-a-phenylbenzyl)oxy] ethylamine citrate is a centrally acting skeletal muscle relaxant that inhibits certain neurons in the brain’s neurological system, hence preventing the generation of impulses in the somatic nerves (Fig. 1). The synergistic effect of combining an analgesic medication with a skeletal muscle relaxant surpasses the individual efficacy of either agent alone [7].

Through a literature study, it has been found that the determination of ASP and CAF in their binary combination was accomplished using electrochemical techniques [8, 9] and HPLC methods [10, 11]. Moreover, the existing literature has shown that there have been limited approaches used to analyze the combination of ASP, CAF, and ORP in their ternary mixes and pharmaceutical formulations. The determination was conducted using the spectrophotometric technique [12], the TLC method [13], and the HPLC methods [14, 15].

UHPLC, TLC, and HPLC are analytical approaches that provide enhanced sensitivity and selectivity. Nevertheless, these approaches are distinguished by their heightened intricacy, elevated costs associated with equipment care, and analysis duration. Furthermore, it is imperative to do sample purifying prior to the injection. UV-spectrometry is an inexpensive, quick, and sensitive analytical instrument; nevertheless, the direct UV-spectrophotometric methods are unsuitable for analyzing multicomponent drug formulations as a result of spectral overlap and lack of specificity. Chemometric is currently a captivating area of study that combines mathematical and computational methods to effectively analyze complicated mixtures and separate overlapping spectra by eliminating noise in the recorded signal [16].

Recently, the scientific sector has been actively adopting environmental chemistry and green analytical chemistry (GAC) ideas into their work methodologies. Consequently, a set of criteria was developed to measure ecological compatibility and sustainability in order to assess the analytical methodologies [17, 18]. Two metrics, GAPI and AGREE, were used in this research to evaluate the proposed method’s sustainable profile. Greenness of the procedure was shown by the findings [19, 20]. Additionally, the procedure’s practicability was evaluated using the newly introduced Blue Applicability Grade Index (BAGI) methodology [21].

As far as we know, there is currently no UV-Chemometric technique available for analyzing this ternary combination. The innovation of this study is in the development of a new chemometric approach that utilizes UV-Spectrophotometer equipment to analyze this combination without the need for separation or sample treatment.

The objective of this study is to create efficient and environmentally-friendly UV-chemometric techniques that are cost-effective, precise, rapid, and user-friendly. These methods will be used to analyze ASP, CAF, and ORP in their combined tablet formulation, without any interference. By comparing the results obtained from different sets of data, we will identify the set with the highest predictive capability. The data have been subjected to statistical analysis and then compared and evaluated.

## Experimental

### Apparatus

The JASCO dual beam UV-visible spectrophotometer model V-630, manufactured in Tokyo, Japan, was used in conjunction with an ACER-compatible computer running spectra management II software. The instrument’s spectral slit width was 2 nm and it could scan at different speeds up to 8000 nm/min. Every measurement has been conducted in a 1 cm quartz cell at normal temperature, spanning a wavelength range of 200 to 400 nm.

### Software

The chemometric analysis was performed using the MATLAB^®^ 7.0.1 software (https://www.mathworks.com/products/matlab.html). The statistical analysis was performed utilizing the PASW Statistics 18^®^ software (http://www.spss.com.hk/statistics/).

### Materials and reagents

#### Pure standards

ASP was obtained from El-Nasr Pharmaceutical Co., Abu Zaabal, Cairo, Egypt. ORP was obtained as a gift from EIPICO, located in the 10th of Ramadan city, Egypt. Their purity was reported to be 99.70%. CAF was obtained from LABORT FINE CHEM, located in 602/A, President Plaza, Near RTO, Ring Road, Surat- 395,001, Gujarat, India, and its purity was reported to be 99.00%.

#### Pharmaceutical formulations

Relatic^®^ tablets were obtained from the market (label claim: ASP 770 mg, CAF 60 mg, and ORP 50 mg) manufactured by Sigma for Pharmaceutical International (SPI), Egypt for Horizone Pharma, Egypt.

#### Solvents

HPLC grade Methanol (LiChrosolv, Merck KGaA, Germany). All of measurements have been carried out by using 90% Methanol (HPLC grade methanol: Distilled water 9:1).

#### Standard solutions Preparation

**Fig. 2:**
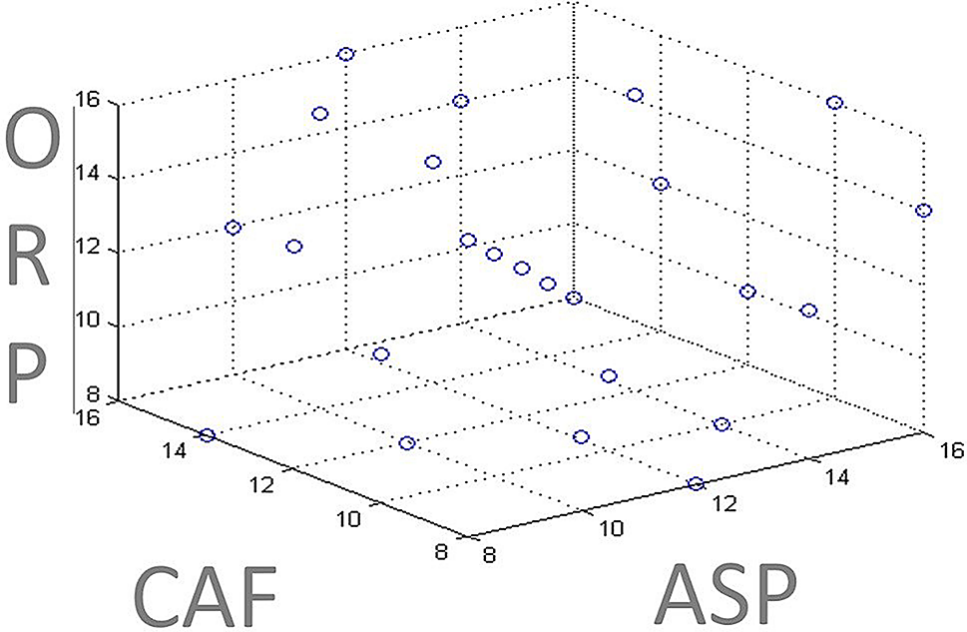
Concentration set design for ASP, CAF & ORP for CLS, PLS and PCR methods

ASP, CAF, ORP stock standard solutions of 1 mg/mL have been prepared in 90% methanol. All working standard solutions of 50 µg/mL have been prepared by dilution from the stock solution with 90% methanol. 25 mixture solutions of ASP, CAF & ORP in the range of 4–25 µg/mL for ASP, 5–35 µg/mL for CAF and 5–50 µg/mL for ORP in the same solvent have been symmetrically prepared from the previous stock solutions respectively and the concentration set design was demonstrated in Fig. 2.

### Calibration set

17 synthetic mixtures in the range of 4–25 µg/mL for ASP, 5–35 µg/mL for CAF and 5–50 µg/mL for ORP were prepared as a training set according to the multifactor and the multilevel design [22] in 10 mL volumetric flasks. Calibration set was chosen according to stratified random sampling technique [23]. UV absorption spectra in its raw form and in its manipulated spectral data sets were used to construct the chemometric models of CLS, PLS and PCR.

### Prediction set

8 synthetic mixtures in the same range of calibration set of the three drugs were also prepared as a validation set according to the multifactor and the multilevel design [22] in 10 mL volumetric flasks to evaluate the accuracy and precision of the constructed models.

### Data preprocessing

Absorption spectra were scanned from 200 to 400 nm while the region 200–215 nm was neglected to avoid noise interference. Several wavelengths have been tried and the wavelength range of 260–285 nm was chosen due to its superior and accurate results over other ranges.

The first and Second derivatives of the absorption spectra were processed before building some models to assess their effect on the validation of the analysis. Ratio spectra were calculated using divisors of ASP (25ug), CAF (25ug), and ORP (25ug), separately then the analysis was continued using only CAF (25ug) as a divisor and its ratio derivatives as it gave more precise values than ASP and ORP.

### Application to pharmaceutical formulation

10 Tablets of Relatic^®^ were weighed and crushed then an amount equivalent to 385 mg ASP, 30 mg CAF and 25 mg ORP in each tablet was transferred into a 50 mL volumetric flask (equivalent to 7700 µg/mL ASP, 600 µg/mL CAF and 500 µg/mL ORP) and diluted with 90% methanol as follow: First, 35 mL of 90% methanol were added and sonicated then filtered and dilution was carried out to the mark. Second, 1 mL of the dilution was transferred into a 100 mL volumetric flask to give a concentration equivalent to 77 µg/mL ASP, 6 µg/mL CAF, and 5 µg/mL ORP. Third, any further dilutions were done in 10 mL volumetric flasks (Sample enrichment must be done to CAF and ORP) and treated in the same way as described under the proposed methods.

## Results and discussion

### Method optimization

**Fig. 3:**
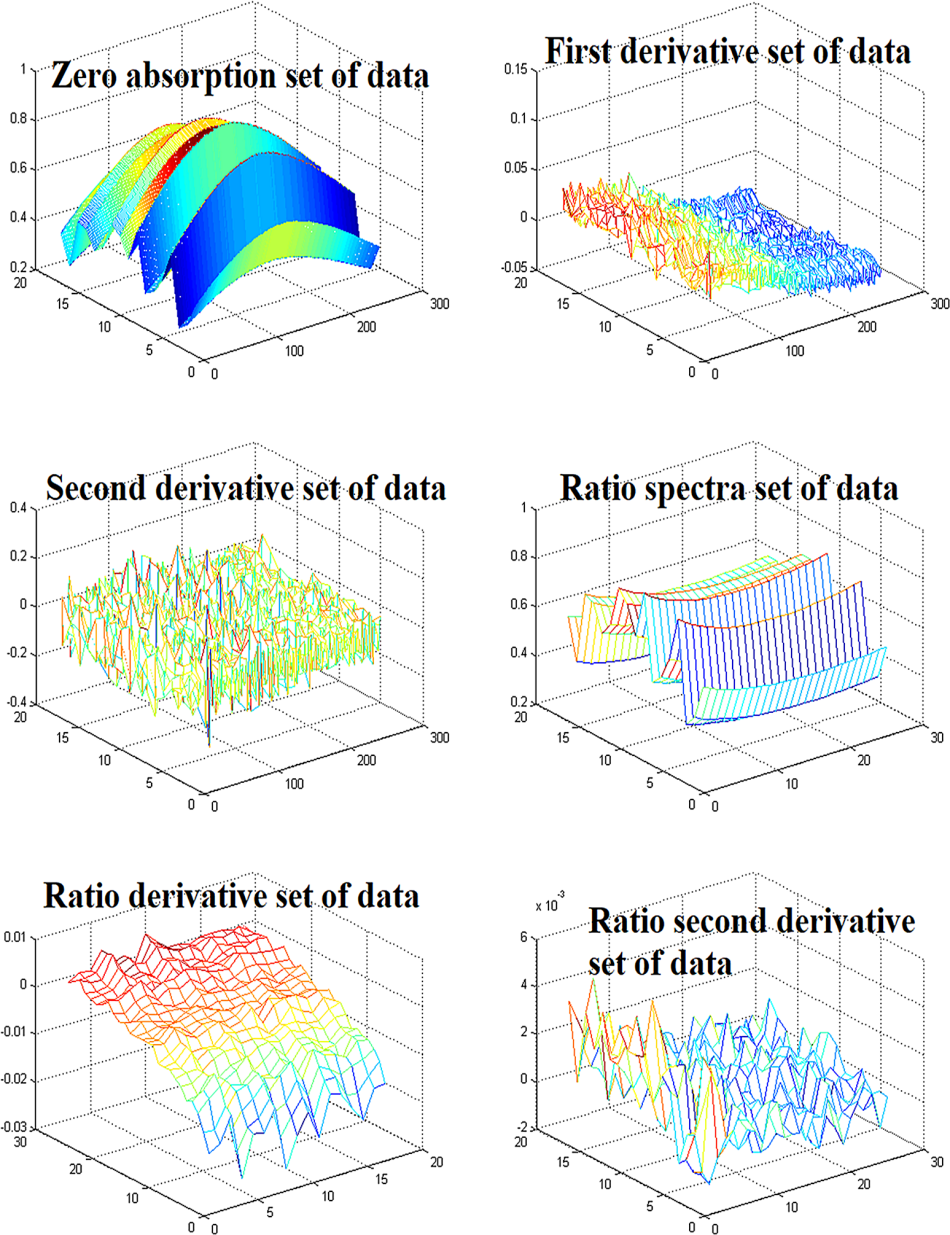
Different sets of data used for construction of CLS, PLS and PCR models represent the range of absorption for each set of data

**Fig. 4:**
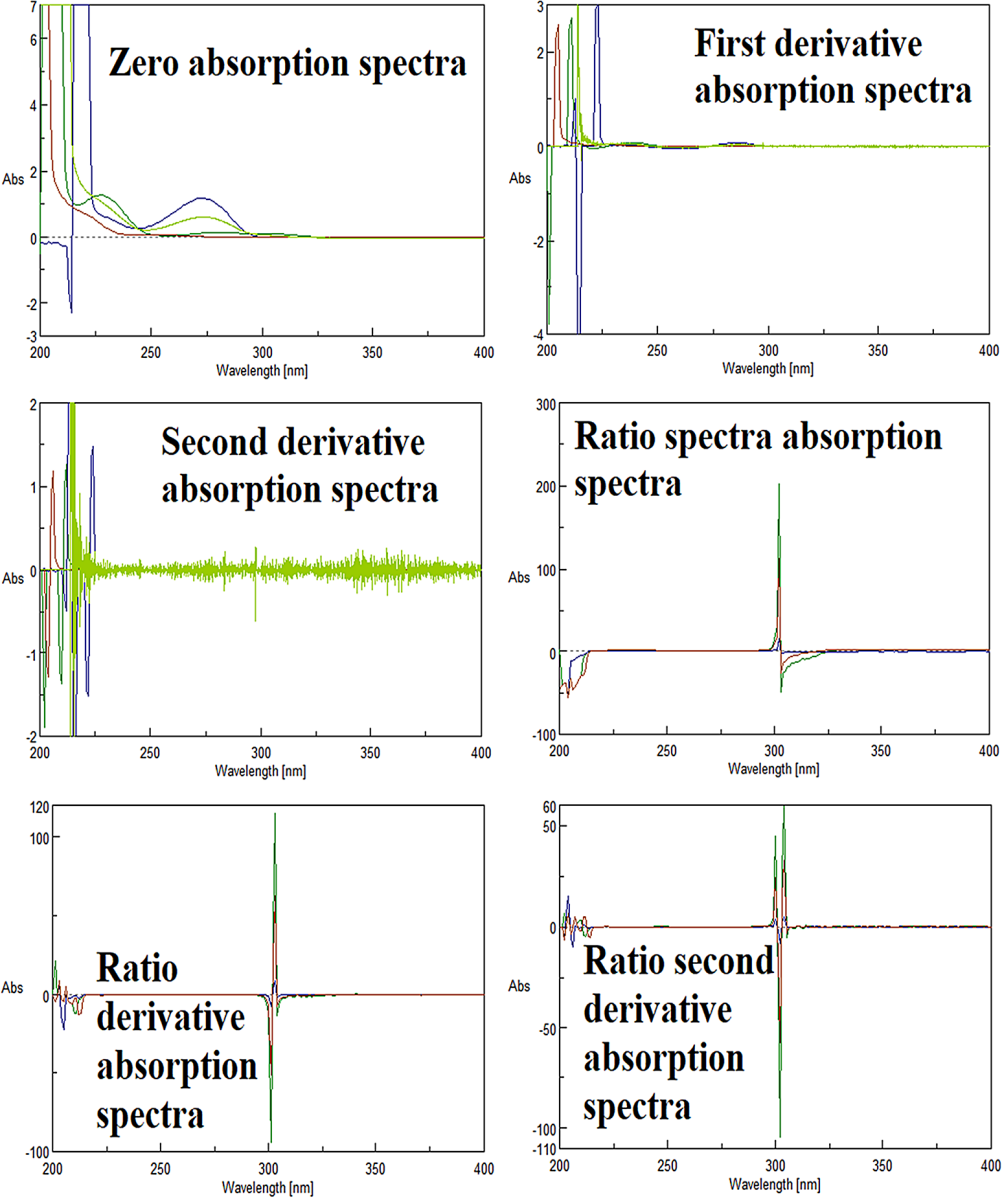
Different absorption spectra of ASP (25ug), CAF (25ug) and ORP (25ug) and their mixture (12ug each) represent the range of absorption for each set of data

Trials of three simple chemometric methods CLS, PLS & PCR have been used for simultaneous determination of ASP, CAF & ORP in their pharmaceutical dosage form. Absorption spectra in their raw form and in their manipulated forms (First derivative, Second derivative, Ratio spectra, First derivative of ratio spectra, and Second derivative of ratio spectra) to make different sets of data (Fig. 3) were used to build CLS, PLS and PCR models in the range of 260–285 nm. 90% Methanol (HPLC grade methanol: Distilled water 9:1) was used as a solvent in which all drugs showed good solubility. Figure 4 displays the absorption spectra of Zero, First derivative, Second derivative, Ratio spectra, First derivative of ratio spectra, and Second derivative of ratio spectra of ASP, CAF & ORP, and their mixture. CLS, PLS, and PCR models were constructed by using a calibration set consisting of different ratios of ASP, CAF & ORP as shown in Table 1.

**Table 1:** Chemometric design for calibration and validation sets for ASP, CAF & ORP

Set	Calibration set			Set	Calibration set			Set	Validation set		
Mix No.	ASP	CAF	ORP	Mix No.	ASP	CAF	ORP	Mix No.	ASP	CAF	ORP
**C1**	12	12	12	**C10**	12	16	16	**V1**	8	16	10
**C 2**	12	8	8	**C11**	16	16	8	**V 2**	10	16	12
**C 3**	8	8	16	**C12**	8	14	8	**V 3**	10	14	16
**C 4**	16	10	16	**C13**	8	12	14	**V 4**	16	14	12
**C 5**	16	12	10	**C14**	12	14	14	**V 5**	16	8	14
**C 6**	12	10	10	**C15**	14	14	10	**V 6**	14	8	12
**C 7**	10	10	14	**C16**	10	8	10	**V 7**	14	10	8
**C 8**	14	16	14	**C17**	10	12	8	**V 8**	8	10	12
**C 9**	14	12	16								

• Concentrations used for ASP, CAF and ORP are 8 ug, 10 ug, 12 ug, 14 ug and 16 ug for each

**Fig. 5:**
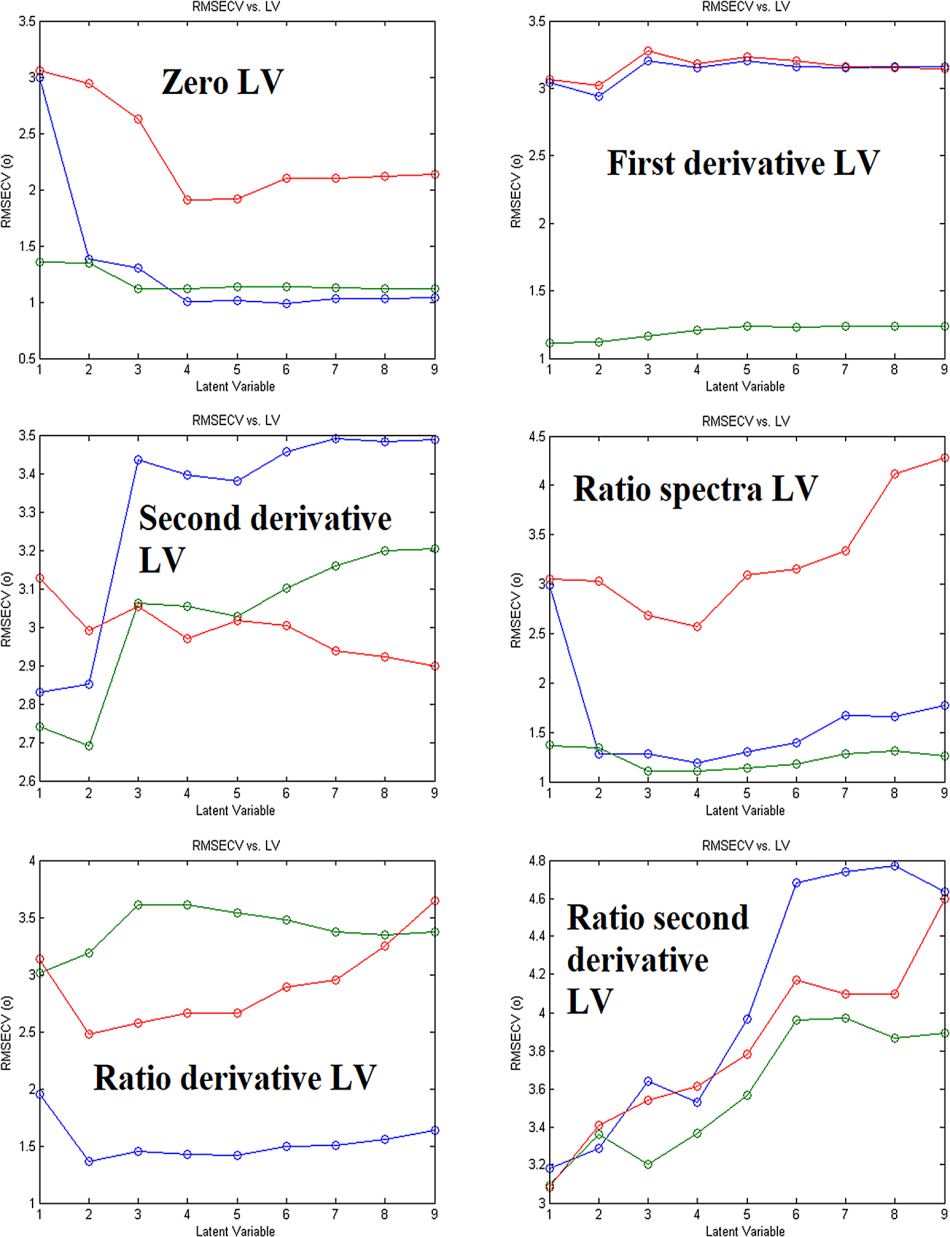
Different Latent Variables (RMSECV vs. LV) for different sets of data for construction of PLS models (Blue, green and red lines represent ASP, CAF and ORP, respectively)

**Fig. 6:**
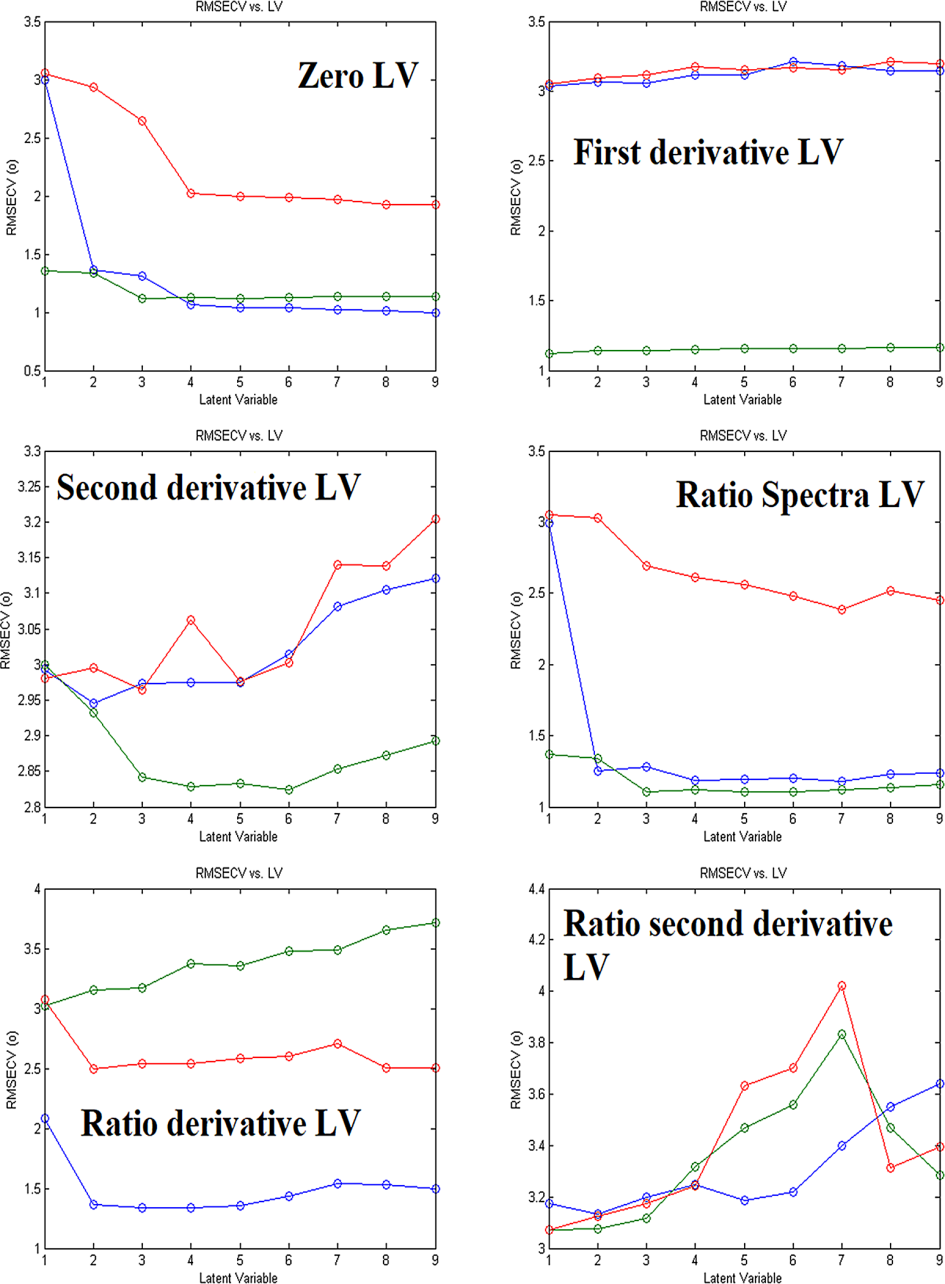
Different Latent Variables (RMSECV vs. LV) for different sets of data for construction of PCR models (Blue, green and red lines represent ASP, CAF and ORP, respectively)

Cross-validation and Scaling were carried out on the calibration set by leaving out one at a time cross-validation and mean center scaling for PLS and PCR models. The number of latent variables is varied from one model to another. Wavelength range from 260 to 285 nm with Δ_λ_ = 0.1 nm for zero, first, and second derivative and Δ*λ* = 1 nm for Ratio spectra and its derivatives were used in all measurements as it is found to give better and more accurate results. Parameters used in the construction of PLS and PCR models are demonstrated in Table 2. The optimal number of latent variables is different from one model to another and is demonstrated for PLS in Fig. 5 and for PCR in Fig. 6.

**Table 2:** Chemometric parameters used for construction of PLS & PCR models

Method	Range (nm)	Interval (nm)	Scaling	Cross Validation
Zero	260–285	0.1	Mean center	Leave one out
First derivative	260–285	0.1	Mean center	Leave one out
Second derivative	260–285	0.1	Mean center	Leave one out
Ratio spectra	260–285	1	Mean center	Leave one out
Ratio derivative	260–285	1	Mean center	Leave one out
Ratio second derivative	260–285	1	Mean center	Leave one out

### Method validation

The Validation of CLS, PLS, and PCR models were calculated by the analysis of their predictive ability on the validation (prediction) set for assessment of the accuracy and precision. The predicted values and actual values of both calibration and validation sets were compared then the predicted residual error sum of squares (PRESS) and root mean square error of prediction (RMSEP) were calculated for various models as follows:

PRESS = Calculate the difference between expected values and predicted values for all the samples and square them then sum them together.

RMSEP = Divide PRESS by number of mixtures and calculate the root of the resulted value.

Results for different sets of data by using PLS are shown in Table 3.

**Table 3:** Results obtained from PLS models for determination of ASP, CAF & ORP in calibration and validation sets

Spectra order		Zero			First derivative			Second derivative			Ratio spectra			Ratio derivative			Ratio 2^nd^ derivative		
PLS	Parameter	ASP	CAF	ORP	ASP	CAF	ORP	ASP	CAF	ORP	ASP	CAF	ORP	ASP	CAF	ORP	ASP	CAF	ORP
Calibration set	Mean	99.78	100.32	99.51	99.81	100.00	100.20	99.97	100.31	100.56	98.02	103.50	102.55	98.72	104.04	103.45	99.63	106.17	105.22
	RMSEP	0.2304	0.2271	0.2571	0.2303	0.1699	0.2356	0.3074	0.3154	0.3228	3.4975	2.4447	3.1148	3.6164	2.6107	3.1372	2.9212	2.7890	2.9719
	PRESS	0.9023	0.8771	1.1237	0.9019	0.4905	0.9432	1.6063	1.6910	1.7711	207.9551	101.5981	164.9332	222.3275	115.8640	167.3122	145.0681	132.2380	150.1522
Validation set	Mean	100.58	99.44	101.18	100.54	100.02	99.80	100.33	99.60	99.12	117.87	104.36	105.69	116.20	102.68	104.09	118.97	104.83	107.89
	RMSEP	0.1504	0.1503	0.3161	0.1743	0.1324	0.2590	0.2728	0.2459	0.2179	3.3262	4.1954	1.8864	2.9421	3.4651	1.7904	2.8550	2.6931	2.4248
	PRESS	0.1808	0.1806	0.7996	0.2431	0.1402	0.5368	0.5954	0.4837	0.3798	88.5084	140.8140	28.4672	69.2457	96.0542	25.6439	65.2098	58.0221	47.0382

Unfortunately, CLS and PCR gave inaccurate results and as such, they would not be used in determination of this ternary mixture unlike PLS which gave very accurate values as shown in Tables S1–S4.

In respect to PLS, Zero absorption spectra, First derivative spectra, and Second derivative spectra can be used for the determination of ASP, CAF & ORP in which Zero absorption spectra have the most powerful prediction for ASP and First derivative spectra have the most powerful prediction for both CAF & ORP while Ratio spectra, Ratio derivative spectra and Ratio second derivative spectra can’t be used for determination of ASP, CAF & ORP.

Although the Raw data set (Zero spectra) is the simplest method but manipulation of the spectra to have different sets of data led to a great difference with improving the results. Although the ratio spectra set of data requires an extra process before carrying out the measurements, the First and Second derivatives of ratio spectra sets of data require more work as they need more extra process than the ratio spectra.

## Application to pharmaceutical formulation

The proposed chemometric method (PLS) was successfully applied for determination of ASP, CAF & ORP in their tablet formulation (Relatic^®^ tablets). The results were in the acceptable range concurrent with the labeled amounts. The standard addition technique (a known amount of standard was added to the formulation and then measured) was applied for accuracy and demonstrated that no interference of the excipients was observed (Table 4).

**Table 4:** Pharmaceutical Preparation (Relatic ^®^ tablets) and standard addition results from using PLS chemometric models

Spectra order		Zero			First derivative			Second derivative			Ratio spectra			Ratio derivative			Ratio 2nd derivative		
PLS	Parameter	ASP	CAF	ORP	ASP	CAF	ORP	ASP	CAF	ORP	ASP	CAF	ORP	ASP	CAF	ORP	ASP	CAF	ORP
Pharmaceutical formulation	Mean	100.02	100.03	100.08	100.03	100.01	99.99	100.03	100.12	99.81	103.75	102.42	103.66	103.49	105.40	102.75	103.74	103.42	108.93
	SD	0.05	0.14	0.19	0.15	0.04	0.04	0.42	0.42	0.62	6.37	9.15	7.64	9.98	9.21	16.87	22.74	15.66	16.23
Standard addition technique	Mean	100.04	100.03	100.12	100.01	99.99	100.05	99.52	99.85	99.93	102.75	102.33	103.71	107.71	105.77	102.86	108.45	102.91	106.37
	SD	0.08	0.16	0.12	0.18	0.08	0.07	0.52	0.41	0.75	8.22	8.64	6.20	11.07	13.98	13.45	18.46	15.16	8.94

## Assessment of the proposed approaches level of eco-friendliness

**Fig. 7:**
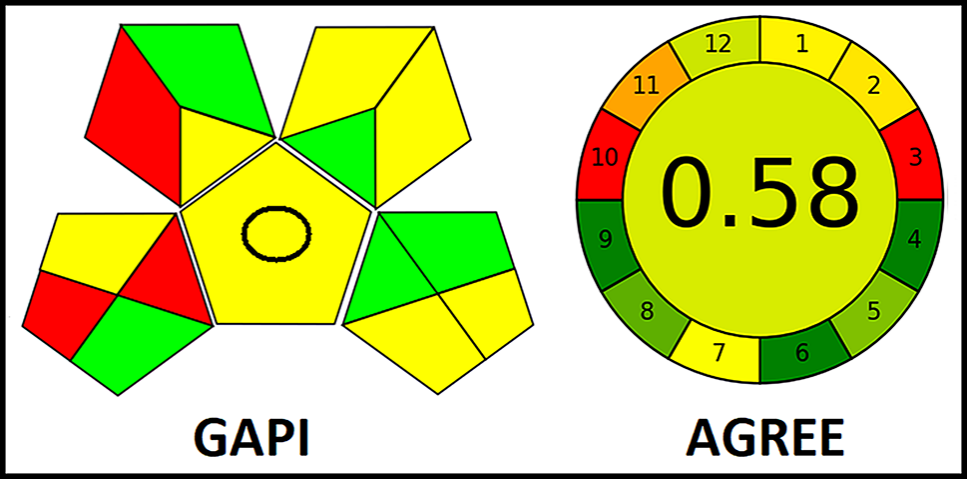
Environmental friendliness evaluation of the technique

Green analytical chemistry is concerned with determining how to put a numerical value on the many environmental impacts of chemical analysis. Researchers and practitioners in the chemical sciences have enormous hurdles in determining whether chemical processes are environmentally friendly and in avoiding unintended consequences. The level of greenness of the intended method was assessed using two contemporary metrics, namely GAPI and AGREE. The use of GAPI here allows for a comprehensive representation of the established technique (https://cdn.mostwiedzy.pl/73/48/4c/f1/0_202010301350285966291_FME/gapi-chart-generator-sfx.exe). The influence on the environment is quantified using five pentagrams. The objects are categorized into three color-coded groups: green, yellow, and red, indicating low, medium, and significant environmental consequences, respectively [24–26]. AGREE (http://mostwiedzy.pl/AGREE) is a cutting-edge measure designed to evaluate the environmental and occupational hazards associated with the analytical process, focusing on 12 main factors. The final score is a fractional value ranging from zero to one, which is located in the center of the AGREE icon. The resulting color in the center is a blend of colors that represent the performance of the 12 AGREE pictogram areas. The optimal approach is assigned a score of 1 and is represented by a dark green color. Hence, the AGREE metric is regarded as user-friendly, comprehensive, easily applicable, and highly efficient [27, 28]. The results shown in Fig. 7 illustrate the effectiveness of an environmentally friendly approach, as indicated by the computed GAPI pictogram and the satisfactory AGREE score of 0.58.

## Assessment of the practicability of the proposed techniques

**Fig. 8:**
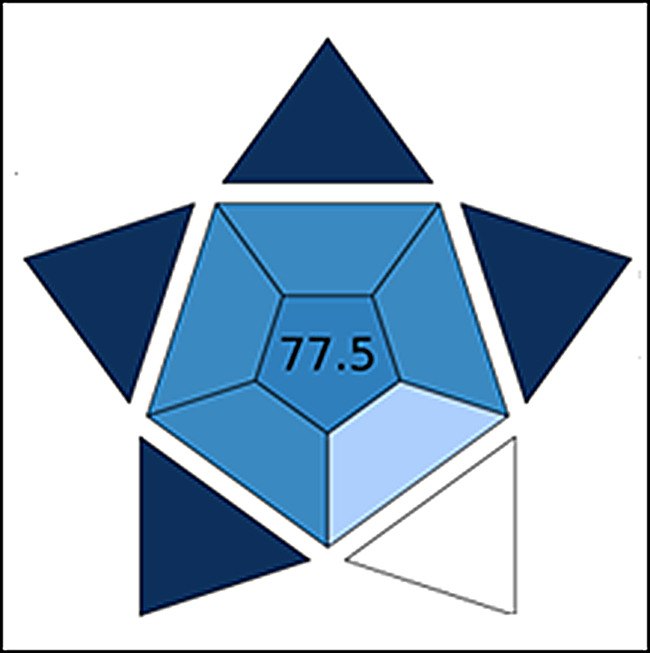
Evaluating the practicality of the proposed strategy utilizing BAGI tools

The Blue Applicability Grade Index (BAGI) is proposed as an innovative tool for measuring the applicability of an analytical approach (git.pg.edu.pl/p174235/bagi). Ten essential variables are evaluated by this tool: analysis kind, hourly sample analysis productivity, analytical technique, reagents and materials used, needed instrumentation, capacity to treat samples concurrently, the necessity of preconcentration, automating level, sample setup method, size of sample, and the capability for concurrent determination of analytes. A visual depiction of an asteroid and its associated score is generated by evaluating the aforementioned characteristics. The following color scheme is used to symbolize different degrees of acceptance: white for no acceptance, light blue for limited acceptance, blue for moderate acceptance, and dark blue for ideal acceptance. To be considered “practical,” it is advised that the technique reaches a minimum of 60 points [21, 29]. The assigned BAGI rating of 77.5 indicates the strategy’s level of practicality (Fig. 8).

## Statistical analysis

Statistical comparison between PLS in zero absorption set of data and the reference reported method [12] was carried out and statistical comparison between the proposed chemometric methods in different sets of data has been carried out by One-way ANOVA method through PASW statistics 18^®^ software program. The calculated F values were less than the theoretical ones in both analysis indicating that there was no significant difference between the proposed methods and no significant difference between different sets of data of the proposed method as reported in Tables 5 and 6.

**Table 5:** Statistical comparison of the results obtained by PLS and the reported method using One-way ANOVA

Models	Drugs		Sum of Squares	df	Mean Square	F	Sig.
PLS	ASP	Between Groups	0.019	1	0.019	0.032	0.867
		Within Groups	2.431	4	0.608		
		Total	2.450	5			
	CAF	Between Groups	0.608	1	0.608	1.757	0.256
		Within Groups	1.385	4	0.346		
		Total	1.993	5			
	ORP	Between Groups	0.032	1	0.032	0.083	0.788
		Within Groups	1.561	4	0.390		
		Total	1.594	5			

**Table 6:** Statistical comparison of the results obtained by the proposed methods in different sets of data using One-way ANOVA

Models	Drugs		Sum of Squares	df	Mean Square	F	Sig.
PLS	ASP	Between Groups	59.606	5	11.921	0.109	0.988
		Within Groups	1315.070	12	109.589		
		Total	1374.676	17			
	CAF	Between Groups	75.240	5	15.048	0.218	0.948
		Within Groups	827.777	12	68.981		
		Total	903.016	17			
	ORP	Between Groups	186.335	5	37.267	0.368	0.861
		Within Groups	1213.590	12	101.132		
		Total	1399.925	17			

## Conclusion

A UV-Chemometric PLS technique can be used for simultaneous determination of ASP, CAF & ORP in their mixture and pharmaceutical formulation. By applying different sets of data, we can deduce that different prediction powers are obtained. Statistical comparison showed that there was no significant difference neither between the proposed methods nor between different sets of data of the proposed method. The study of the environmental sustainability of the technique using several greenness appraisal methods has revealed that the suggested methodology is eco-friendly. The assessment of the BAGI methodology also shows that the newly designed method is practicable.

## Electronic supplementary material

Below is the link to the electronic supplementary material.

Supplementary Material 1

## Data Availability

The data used and/or analyzed during this study are available from the corresponding author on a reasonable request.

## References

[1] Gebke KB, McCarberg B, Shaw E, Turk DC, Wright WL, Semel D. A practical guide to recognize, assess, treat and evaluate (RATE) primary care patients with chronic pain. Postgrad Med. 2023;135:244–53. 10.1080/00325481.2021.2017201. 35060834 10.1080/00325481.2021.2017201

[2] Bhattacherjee A, Zhang C, Watson BR, Djekidel MN, Moffitt JR, Zhang Y. Spatial transcriptomics reveals the distinct organization of mouse prefrontal cortex and neuronal subtypes regulating chronic pain. Nat Neurosci. 2023;26:1880–93. 10.1038/s41593-023-01455-9. 37845544 10.1038/s41593-023-01455-9 PMC10620082

[3] Gaskin DJ, Richard P. The economic costs of pain in the united States. J Pain. 2012;13:715–24. 10.1016/j.jpain.2012.03.009. 22607834 10.1016/j.jpain.2012.03.009

[4] Pergolizzi Jr JV, Magnusson P, Raffa RB, LeQuang JA, Coluzzi F. Developments in combined analgesic regimens for improved safety in postoperative pain management. Expert Rev Neurother. 2020;20:981–90. 10.1080/14737175.2020.1806058. 32749896 10.1080/14737175.2020.1806058

[5] Patrono C. Fifty years with aspirin and platelets. Br J Pharmacol. 2023;180:25–43. 10.1111/bph.15966. 36189951 10.1111/bph.15966 PMC10099789

[6] Wabaidur SM, Alothman ZA, Khan MR. A rapid method for the simultaneous determination of L -ascorbic acid and acetylsalicylic acid in aspirin C effervescent tablet by ultra performance liquid chromatography – tandem mass spectrometry. Spectrochim Acta Part Mol Biomol Spectrosc. 2013;108:20–5. 10.1016/j.saa.2013.01.070. 10.1016/j.saa.2013.01.070 23454710

[7] Sebaiy MM, El-Adl SM, Mattar AA. Different techniques for overlapped UV spectra resolution of some co-administered drugs with Paracetamol in their combined pharmaceutical dosage forms. Spectrochim Acta - Part Mol Biomol Spectrosc. 2020;224:117429. 10.1016/j.saa.2019.117429. 10.1016/j.saa.2019.117429 31394394

[8] Ghadimi H, Tehrani RMA, Jefrey W, Junaida N, Aziz A, Mohamed N, Ab S. Electrochemical determination of aspirin and caffeine at MWCNTs-poly-4-vinylpyridine composite modified electrode. J Taiwan Inst Chem Eng. 2016;65:101–9. 10.1016/j.jtice.2016.05.043.

[9] Goyal RN, Bishnoi S, Agrawal B. Electrochemical sensor for the simultaneous determination of caffeine and aspirin in human urine samples. J Electroanal Chem. 2011;655:97–102. 10.1016/j.jelechem.2011.03.008.

[10] Bharate SS, Bharate SB. Spectrophotometric and chromatographic determination of acetylsalicylic acid and caffeine in pure and in tablet dosage form. J Adv Sci Res. 2012;3:73–81.

[11] Ferguson GK. Quantitative HPLC analysis of an analgesic / caffeine formulation: determination of caffeine. J Chem Educ. 1998;75:467–9. 10.1021/ed075p467.

[12] Abdelrahman MM. Selective spectrophotometric methods for determination of ternary mixture with overlapping spectra: A comparative study, spectrochim. Acta part A mol. Biomol Spectrosc. 2014;124:389–96. 10.1016/j.saa.2014.01.020. 10.1016/j.saa.2014.01.020 24508877

[13] Gamal M, Ali NW, Elghobashy MR, Abdelkawy M. Simultaneous determination of ternary mixture of aspirin, caffeine and orphenadrine citrate by simple RP-TLC spectrodensitometric method. Br J Pharm Res. 2017;14:1–11. 10.9734/BJPR/2016/31194.

[14] Pai SP, Gaude S, Palekar A. RP-HPLC method development and validation for simultaneous Estimation of aspirin, caffeine and orphenadrine citrate in tablet formulation. Int J Sci Res. 2013;5:1170–3.

[15] Darwish K, Salama I, Mostafa S, El-sadek M. Validated stability-indicating reversed-phase-HPLC method for simultaneous determination of orphenadrine citrate, caffeine and aspirin. Chem Pharm Bull. 2012;60:1426–36. 10.1248/cpb.c12-00596. 10.1248/cpb.c12-00596 23124566

[16] Sayed RA, Ibrahim AE, Sharaf YA. Chemometry-assisted UV‐spectrophotmetric methods for the simultaneous determination of paritaprevir, Ritonavir, and ombitasvir in their combined tablet dosage forms: A comparative study. J Chemom. 2021;35:e3339. 10.1002/cem.3339.

[17] Shi M, Zheng X, Zhang N, Guo Y, Liu M, Yin L. Overview of sixteen green analytical chemistry metrics for evaluation of the greenness of analytical methods. TrAC Trends Anal Chem. 2023;166:117211. 10.1016/j.trac.2023.117211.

[18] Sajid M, Płotka-Wasylka J. Green analytical chemistry metrics: A review. Talanta. 2022;238:123046. 10.1016/j.talanta.2021.123046. 34801903 10.1016/j.talanta.2021.123046

[19] Pena-Pereira F, Wojnowski W, Tobiszewski M. AGREE - Analytical greenness metric approach and software. Anal Chem. 2020;92:10076–82. 10.1021/acs.analchem.0c01887. 32538619 10.1021/acs.analchem.0c01887 PMC7588019

[20] Płotka-Wasylka J. A new tool for the evaluation of the analytical procedure: green analytical procedure index. Talanta. 2018;181:204–9. 10.1016/j.talanta.2018.01.013. 29426502 10.1016/j.talanta.2018.01.013

[21] Manousi N, Wojnowski W, Płotka-Wasylka J, Samanidou V. Blue applicability grade index (BAGI) and software: a new tool for the evaluation of method practicality. Green Chem. 2023;25:7598–604. 10.1039/D3GC02347H.

[22] Brereton R.G. Introduction to multivariate calibration in analytical chemistry. Analyst. 2000;2125–54. 10.1039/b003805i.

[23] Reitermanov Z. Data Splitting, WDS’10 Proc. Contrib. Pap. 2010;31–36.

[24] Eissa MS, Darweish E. Insights on ecological spectroscopic techniques recently adopted for pharmaceutical analysis: A comprehensive review from the perspective of greenness assessment metrics systems application. TrAC Trends Anal Chem. 2023;170:117435. 10.1016/j.trac.2023.117435.

[25] El-Sayed HM, Abdellatef HE, Hendawy HAM, El-Abassy OM, Ibrahim H. DoE-enhanced development and validation of eco-friendly RP-HPLC method for analysis of Safinamide and its precursor impurity: QbD approach. Microchem J. 2023;108730. 10.1016/j.microc.2023.108730.

[26] Ibrahim H, El-Abassy OM, Abdellatef HE, Hendawy HAM, El-Sayed HM. Simultaneous analysis of two drugs used as supportive treatment for COVID-19: comparative statistical studies and analytical ecological appraisal. BMC Chem. 2022;16:1–15. 10.1186/s13065-022-00860-8. 36167604 10.1186/s13065-022-00860-8 PMC9514717

[27] Soliman SS, Mahmoud AM, Elghobashy MR, Zaazaa HE, Sedik GA. Eco-friendly electrochemical sensor for determination of conscious sedating drug midazolam’’based on Au-NPs@ silica modified carbon paste electrode. Talanta. 2024;267:125238. 10.1016/j.talanta.2023.125238. 37774450 10.1016/j.talanta.2023.125238

[28] El-Maghrabey M, Magdy G, Hashem HM, Amin M, Elgaml A, Radwan A, El-Sherbeny M, El-Shaheny R. Comprehending COVID-19 diagnostic tests and greenness assessment of its reported detection methods. TrAC Trends Anal Chem. 2023;169:117379. 10.1016/j.trac.2023.117379.

[29] El Hamd MA, Soltan OM, Abdelrahman KS, Fouad A, Saleh SF, Obaydo RH, Sallam S, Alshehri S, Mahdi WA, Hamad AA. Roth’s switch-on fluoremetric probe for green tracking and quantifying of 1.4-dihydropyridine medication: evaluation of greenness, whiteness, and blueness. Sustain Chem Pharm. 2023;36:101294. 10.1016/j.scp.2023.101294.

