# Supplementary material for: Development of UV-Chemometric techniques for resolving the overlapped spectra of aspirin, caffeine and orphenadrine citrate in their combined pharmaceutical dosage form
Source: BMC Chem. 2025 Mar 20;19(1):75. doi: 10.1186/s13065-025-01429-x (PMC11927279; doi:10.1186/s13065-025-01429-x)
Supplement: Supplementary file 1 — Supplementary Material 1 [file 13065_2025_1429_MOESM1_ESM.docx]

**Table S1**  Results obtained from CLS models for determination of ASP, CAF & ORP in calibration and validation sets.

| **Spectra order** | | **Zero** | | | **First derivative** | | | **Second derivative** | | | **Ratio spectra** | | | **Ratio derivative** | | | **Ratio 2^nd^ derivative** | | |
| --- | --- | --- | --- | --- | --- | --- | --- | --- | --- | --- | --- | --- | --- | --- | --- | --- | --- | --- | --- |
| **CLS** | **Parameter** | **ASP** | **CAF** | **ORP** | **ASP** | **CAF** | **ORP** | **ASP** | **CAF** | **ORP** | **ASP** | **CAF** | **ORP** | **ASP** | **CAF** | **ORP** | **ASP** | **CAF** | **ORP** |
| **Calibration set** | Mean | 98.05 | 100.18 | 97.13 | 100.35 | 100.04 | 101.86 | 105.33 | 107.07 | 107.18 | 96.94 | 103.88 | 99.14 | 97.59 | 107.40 | 103.58 | 100.97 | 103.63 | 108.29 |
|  | RMSEP | 1.1736 | 1.1199 | 3.1633 | 1.3412 | 1.1367 | 1.5785 | 2.2595 | 1.9019 | 2.3740 | 3.4873 | 2.9225 | 4.5594 | 3.4886 | 3.3257 | 3.0522 | 6.3638 | 6.7271 | 6.0642 |
|  | PRESS | 23.4146 | 21.3225 | 170.1111 | 30.5809 | 21.9646 | 42.3576 | 86.7930 | 61.4947 | 95.8083 | 206.7468 | 145.2019 | 353.3955 | 206.9013 | 188.0304 | 158.3659 | 688.4588 | 769.3201 | 625.1578 |
| **Validation set** | Mean | 106.77 | 96.66 | 104.84 | 103.77 | 96.88 | 102.21 | 99.34 | 91.95 | 97.16 | 121.08 | 101.71 | 108.59 | 120.45 | 99.41 | 109.76 | 118.60 | 110.51 | 106.77 |
|  | RMSEP | 1.6540 | 0.5767 | 4.3564 | 1.3755 | 0.5082 | 1.5130 | 2.1707 | 1.6343 | 2.0079 | 3.5296 | 4.3175 | 4.6299 | 3.4727 | 4.5018 | 4.4459 | 5.8418 | 5.7503 | 5.0705 |
|  | PRESS | 21.8863 | 2.6602 | 151.8255 | 15.1359 | 2.0664 | 18.3142 | 37.6966 | 21.3664 | 32.2521 | 99.6639 | 149.1247 | 171.4841 | 96.4785 | 162.1260 | 158.1254 | 273.0084 | 264.5308 | 205.6783 |

**Table S2**  Results obtained from PCR models for determination of ASP, CAF & ORP in calibration and validation sets.

| **Spectra order** | | **Zero** | | | **First derivative** | | | **Second derivative** | | | **Ratio spectra** | | | **Ratio derivative** | | | **Ratio 2^nd^ derivative** | | |
| --- | --- | --- | --- | --- | --- | --- | --- | --- | --- | --- | --- | --- | --- | --- | --- | --- | --- | --- | --- |
| **PCR** | **Parameter** | **ASP** | **CAF** | **ORP** | **ASP** | **CAF** | **ORP** | **ASP** | **CAF** | **ORP** | **ASP** | **CAF** | **ORP** | **ASP** | **CAF** | **ORP** | **ASP** | **CAF** | **ORP** |
| **Calibration set** | Mean | 99.40 | 101.50 | 101.07 | 104.74 | 101.21 | 105.90 | 105.76 | 106.23 | 106.70 | 98.09 | 104.26 | 103.69 | 97.59 | 107.40 | 103.58 | 99.68 | 104.34 | 104.51 |
|  | RMSEP | 0.6372 | 0.9273 | 0.9439 | 2.2073 | 1.0067 | 2.3035 | 2.1505 | 1.9766 | 2.3404 | 3.5149 | 2.5655 | 3.0478 | 3.4886 | 3.3257 | 3.0522 | 3.4572 | 2.9546 | 3.0683 |
|  | PRESS | 6.9020 | 14.6185 | 15.1476 | 82.8304 | 17.2302 | 90.2059 | 78.6163 | 66.4187 | 93.1181 | 210.0234 | 111.8881 | 157.9195 | 206.9013 | 188.0304 | 158.3659 | 203.1833 | 148.4090 | 160.0410 |
| **Validation set** | Mean | 102.41 | 98.31 | 101.75 | 104.02 | 99.38 | 101.20 | 103.25 | 99.56 | 100.18 | 117.34 | 103.01 | 105.40 | 118.88 | 102.25 | 106.95 | 119.71 | 107.59 | 109.18 |
|  | RMSEP | 0.6537 | 0.4404 | 1.8585 | 2.7472 | 0.7151 | 2.6313 | 3.2103 | 2.7292 | 2.7306 | 2.8962 | 3.9965 | 2.3941 | 2.9429 | 2.2745 | 2.7494 | 2.6834 | 2.2639 | 2.4698 |
|  | PRESS | 3.4184 | 1.5518 | 27.6324 | 60.3760 | 4.0910 | 55.3906 | 82.4494 | 59.5863 | 59.6491 | 67.1029 | 127.7754 | 45.8549 | 69.2850 | 41.3859 | 60.4716 | 57.6045 | 41.0019 | 48.8001 |

**Table S3**  Pharmaceutical preparation (Relatic^®^ tablets) and standard addition results from using CLS chemometric models.

| **Spectra order** | | **Zero** | | | **First derivative** | | | **Second derivative** | | | **Ratio spectra** | | | **Ratio derivative** | | | **Ratio 2^nd^ derivative** | | |
| --- | --- | --- | --- | --- | --- | --- | --- | --- | --- | --- | --- | --- | --- | --- | --- | --- | --- | --- | --- |
| **PCR** | **Parameter** | **ASP** | **CAF** | **ORP** | **ASP** | **CAF** | **ORP** | **ASP** | **CAF** | **ORP** | **ASP** | **CAF** | **ORP** | **ASP** | **CAF** | **ORP** | **ASP** | **CAF** | **ORP** |
| **Pharmaceutical formulation** | Mean | 120.37 | 119.51 | 125.05 | 120.22 | 119.77 | 122.09 | 121.82 | 119.63 | 124.93 | 130.15 | 128.83 | 122.52 | 141.76 | 123.70 | 135.22 | 153.04 | 128.45 | 136.62 |
|  | SD | 4.89 | 6.64 | 6.14 | 4.66 | 5.40 | 6.07 | 10.57 | 8.29 | 7.02 | 17.00 | 14.63 | 12.33 | 11.21 | 11.10 | 20.82 | 11.47 | 16.37 | 11.56 |
| **Standard addition technique** | Mean | 130.15 | 121.92 | 127.92 | 120.83 | 121.45 | 122.70 | 120.57 | 118.08 | 118.53 | 121.64 | 126.47 | 132.52 | 129.52 | 132.50 | 137.38 | 145.22 | 132.08 | 129.89 |
|  | SD | 5.33 | 13.07 | 5.63 | 6.65 | 3.69 | 4.40 | 10.11 | 9.47 | 7.36 | 13.53 | 15.14 | 13.03 | 18.43 | 11.17 | 19.91 | 9.34 | 10.90 | 17.95 |

**Table S4**  Pharmaceutical preparation (Relatic ^®^ tablets) and standard addition results from using PCR chemometric models.

| **Spectra order** | | **Zero** | | | **First derivative** | | | **Second derivative** | | | **Ratio spectra** | | | **Ratio derivative** | | | **Ratio 2^nd^ derivative** | | |
| --- | --- | --- | --- | --- | --- | --- | --- | --- | --- | --- | --- | --- | --- | --- | --- | --- | --- | --- | --- |
| **PCR** | **Parameter** | **ASP** | **CAF** | **ORP** | **ASP** | **CAF** | **ORP** | **ASP** | **CAF** | **ORP** | **ASP** | **CAF** | **ORP** | **ASP** | **CAF** | **ORP** | **ASP** | **CAF** | **ORP** |
| **Pharmaceutical formulation** | Mean | 114.65 | 118.70 | 115.54 | 116.75 | 113.58 | 117.59 | 124.96 | 122.51 | 126.26 | 127.86 | 125.47 | 127.79 | 132.52 | 132.23 | 134.30 | 138.83 | 137.67 | 137.71 |
|  | SD | 2.03 | 1.14 | 2.79 | 2.26 | 2.41 | 2.63 | 3.32 | 0.92 | 1.36 | 1.62 | 1.56 | 2.01 | 2.04 | 1.25 | 1.41 | 1.23 | 2.09 | 2.89 |
| **Standard addition technique** | Mean | 115.76 | 119.51 | 116.43 | 117.82 | 114.82 | 118.45 | 124.54 | 125.66 | 127.60 | 129.13 | 127.15 | 126.59 | 135.16 | 132.80 | 136.38 | 138.48 | 136.06 | 138.09 |
|  | SD | 2.03 | 0.75 | 2.26 | 2.17 | 2.49 | 2.44 | 1.96 | 1.72 | 0.89 | 1.63 | 2.16 | 3.21 | 1.47 | 2.02 | 1.80 | 3.40 | 2.09 | 1.69 |
